# The impact of time spent working from home on affective commitment in the workplace: The mediating role of social relationships and collective aims

**DOI:** 10.3389/fpsyg.2022.1002818

**Published:** 2023-01-13

**Authors:** Adél Csenge Simon, Gabor Aranyi, Klára Faragó, Orsolya Csilla Pachner, Orhidea Edith Kiss

**Affiliations:** ^1^Doctoral School of Psychology, Eötvös Loránd University, Budapest, Hungary; ^2^Institute of Psychology, ELTE Eötvös Loránd University, Budapest, Hungary; ^3^ELTE Faculty of Education and Psychology, Institute of Education and Psychology at Szombathely, Szombathely, Hungary; ^4^Department of Organisational and Leadership Psychology, ELTE Faculty of Education and Psychology, Budapest, Hungary

**Keywords:** organizational psychology, affective commitment, digitalization, COVID-19, work from home, latent deprivation model

## Abstract

**Introduction:**

Working from home has become increasingly prevalent due to the COVID-19 pandemic, creating new challenges for organizations and employees. According to the latent deprivation model proposed by Jahoda, work provides latent benefits alongside its material rewards, and losing such benefits leads to a decline in well-being. Organizational affective commitment, or affective commitment within the organization, is a prominent concept in organizational psychology that is linked to lower workforce fluctuation and increased work performance. The present research examined the impact of time spent working from home on affective commitment by examining Jahoda’s “latent functions,” including social contact and collective purpose, representing an innovative application of the latent deprivation model in the context of home office.

**Methods:**

Using an online questionnaire, we collected data from 456 participants (239 female and 217 male) who had been employed for at least 2 years and who had spent a proportion of their time working from home in March and April 2021. The data were analyzed using a path model, in which the potential adverse effect of time spent in home office on affective commitment to the workplace was mitigated by latent functions.

**Results:**

Specifically, we found that more time spent in home office was associated with a decrease in social contact, the impact of which on affective commitment was mediated through the perception of collective purpose.

**Discussion:**

Our findings emphasize the role of the latent benefits of work experienced by employees even when working from home, and the role of those benefits in supporting employees’ commitment to the workplace. We argue that a deeper understanding of such factors is vital, as working from home is expected to remain widespread even after the pandemic.

## Introduction

Organizational digitalization has been progressing steadily for decades and has now reached virtually all fields of work, bringing with it a multitude of changes in how tasks are organized and carried out (Cijan et al., 2019). Among these changes, our research focused on the increasingly widespread phenomenon of working from home, which has recently become the predominant model in many workplaces due to the restrictive measures introduced globally as a result of the COVID-19 pandemic (Davis et al., 2020). It is important to note that the pandemic is not responsible for the development of home office, nor for the progress of digitalization; it has merely accelerated their adoption. In 2019, only 5% of employees worked from their homes. However, there was a massive increase in March 2020, when the pandemic-related restrictions were introduced and it became mandatory to work from home. Zoom, for example, which allowed employees to keep in touch with one another, experienced a 67% growth in users in March 2020, while the number of registered daily users of Microsoft Teams grew by 24 million between November 2019 and March 2020 (Leonardi, 2020).

Due to digital developments, a new variety of work organization—encompassing home office and homeworking—has emerged since the 1970s, allowing employees to distance themselves from their offices (Torten et al., 2016). Home office refers to an employee working from home occasionally for a particular reason, while homeworking is a standardized approach whereby an employee works from home permanently, based on the mutual agreement of the employer and employee (Beňo, 2018). Those with the status of jobholder can opt for a work arrangement known as telecommuting, which enables them to perform their tasks from their home, their office, or any other location using their preferred telecommuting platform. This work arrangement allows for schedule flexibility (Ten Brummelhuis et al., 2012). Many different forms of working from home have appeared as a result of the COVID-19 pandemic.

Working from home has numerous benefits as well as drawbacks for employees and organizations. Some of the advantages are obvious, such as reduced stress and less time lost due to commuting, and, in some instances, greater freedom in terms of workday scheduling. Among the disadvantages, however, employees have reported struggling with the limitations of their digital devices and experiencing increased fatigue due to personal interactions being replaced by the need to spend hours in front of their screens (Narbarte et al., 2020).

Drawing on their experiences during the COVID-19 lockdowns, many employees and organizations aim to maintain a work model based on working from home after restrictions are lifted. However, the effects of working from home on the psychological aspects of work remain largely unexplored. Such a fundamental shift in how work is organized is expected to have a profound impact on employees, notably in terms of their work performance (Gibbs et al., 2021), psychological well-being (Howe and Menges, 2021), and commitment to their employers (Wang et al., 2020).

While many questions remain unanswered, numerous studies in the past 2 years have explored how the COVID-19 pandemic and mandatory home office have affected employees. Researchers have endeavored to measure changes in employees’ sleeping habits (Staller and Randler, 2021) and alterations in their well-being, mood, and work performance (Costa et al., 2022). Some studies have concentrated on the environmental psychological aspects of working from home, thus focusing on physical discomfort and mobility problems (Garcia et al., 2022). One longitudinal study showed that, in the examined two-week period, there was an improvement in workplace commitment and in the experience of flow during work from home (Schade et al., 2021). “Flow” is the notion that someone is so absorbed in their activities that they operate at an optimal level, whether they are working or engaged in a leisure activity (Pels et al., 2018). This suggests that although working from home is relatively new, it is not necessarily a bad form of work organization. In our research, we wanted to examine home office work from a new perspective compared to previously published studies: We wanted to study how the functions in Jahoda’s latent deprivation model affect organizational commitment in light of the time spent working from home.

Organizational commitment is one of the most critical organizational variables and has recently inspired a number of studies. Due to changes generated by economic uncertainty, ongoing globalization, digitalization, and greater mobility on the part of younger generations of employees, the development of affective commitment has become essential in every organization (Cohen, 2007; Fornes et al., 2008; Morrow, 2011; Mercurio, 2015). Today, one important management task is to train and retain talented employees, failing which, the organization risks being placed at an economic and strategic disadvantage compared to its competitors. Interestingly, the importance of commitment is emphasized not only by researchers but also by economic journals (Mercurio, 2015). It is not surprising, then, that organizational commitment has engendered such an extensive literature. The Three-Component Conceptualization of Organizational Commitment model (proposed by Allen and Meyer) comprises affective commitment, continuance commitment, and normative commitment. Affective commitment is the emotional connection that ties the individual to the organization and it includes acceptance of the organization’s values and the desire to stay in the organization (Somers, 1995). All three components are vital for a complex image of organizational commitment; however, according to the latest studies and meta-analyzes, affective commitment demonstrates the strongest connections to various organizational psychology variables, for example, absence, fluctuation, performance, organizational citizenship behaviors, and additional workload (Cooper-Hakim and Viswesvaran, 2005; Solinger et al., 2008; Chordiya et al., 2017; Alkhateri et al., 2018; Jang and Kandampully, 2018).

The present research examined the impact of the proportion of working time spent working from home on people’s commitment to the workplace, while simultaneously considering psychological aspects of work that are also potentially influenced by working from home—notably a change in social contact and the perception of collective purpose.

The non-material benefits of work have long been recognized in organizational theory. In his Theory *X* and Theory *Y*, McGregor stated that people are motivated to work not only by their salary but also by a need to satisfy their social requirements (McGregor, 1960). Marie Jahoda’s influential book, published two decades later, elaborated on the latent benefits of work (Jahoda, 1982). This theory is known as the latent deprivation model. Jahoda identified five such latent benefits: time structure, activity, social contact, collective purpose, and status (Jahoda, 1982). It has been widely reported that the termination of latent benefits is associated with a considerable degree of psychological stress and anxiety for the unemployed (Jahoda, 1982; Paul et al., 2009; Paul and Batinic, 2010; Selenko et al., 2011; Muller and Waters, 2012; Aitken et al., 2021). Jahoda’s latent deprivation model is a theoretical framework that explains the decline in psychological well-being among the unemployed and highlights the notion that employees do not work just for their salary but also to fulfill psychological needs (Creed and Macintry, 2001; Muller and Waters, 2012). This model highlights how working from home can give rise to problems in terms of fulfilling the psychological needs of employees. There are, of course, significant differences between working from home and being unemployed (e.g., there is no loss of salary, the most obvious advantage of work). However, there are also some remarkable similarities (e.g., in both cases the individual is able to manage their time, in contrast to traditional offline work where timetables are shaped by the manager and the organization; while social connections become looser in both cases, of course). Furthermore, the latent benefits were developed with respect to offline work, thus anomalies are to be expected when it comes to working from home.

Each function of latent deprivation has been subject to empirical investigation. Most studies have relied on groups of unemployed people, since the model was originally used to determine changes in the well-being of such groups. For example, work requires people not only to organize their time but also to be more active; accordingly, studies have found that unemployed people who spend their free time actively experience greater psychological well-being (Evans and Haworth, 1991; Waters and Moore, 2002). Another benefit of work is that it ties individuals to objectives that reach beyond them and thus supports the experience of meaning, leading to feelings of usefulness. Jahoda called this collective purpose (Jahoda, 1982), which has been found to be connected positively to well-being and negatively to anxiety (Evans and Haworth, 1991). Furthermore, being occupied provides opportunities for employees to develop social links beyond the family, which also contributes to mental health (Paul and Batinic, 2010). Losing one’s job leads to the narrowing of one’s social connections and to a decrease in social interactions. While it cannot be expected that all employees will share a close and friendly bond in the work environment, being in that environment can still broaden the employee’s social circle (Jahoda, 1982). The narrowing of social connections can lead to a decline in well-being and the development of depression (Creed and Klisch, 2005). A more intensive family life is not a substitute for working relationships, due to differences in the diversity and variety of information (Jahoda, 1982).

Although the latent deprivation theory has received strong empirical support in the past four decades, most studies have focused on the unemployed and thus provide limited results for the population at large. Several researchers have linked latent functions to demographic variables (Paul and Batinic, 2010). In comparative studies using the latent deprivation framework not just with unemployed people as participants but also with people who were occupied, retired, students, or homemakers, those with an occupation scored the highest in latent functions, which supports Jahoda’s original assumption that work is best able to provide latent benefits in society (Creed et al., 2003; Paul et al., 2009; Paul and Batinic, 2010). In a recent study, Scheuring and her colleagues compared employees in fixed-term and permanent employment to unemployed people in an international sample and found differences in well-being from an intercultural perspective. However, Jahoda’s latent deprivation theory still had significant explanatory power in terms of the well-being of all three groups (Scheuring, 2020). In a different study, Batinic and his colleagues concluded that there is a significant difference in terms of the latent benefits of work not just between the unemployed and the employed but also between groups of employees based on their status (higher-status employees experienced the latent benefits more readily and at a higher level; Batinic et al., 2010). In their 2010 study, Paul and Batinic measured the emergence of the latent benefits of work in a German representative sample comprising seven different occupational groups, which showed significant differences in terms of the five examined factors. Based on this, the researchers concluded that people experience the latent benefits differently in different working conditions, which in some cases can lead to a decline in mental health (Paul and Batinic, 2010). It has been proven using a four-wave longitudinal study that a decrease in the latent benefits damages psychological well-being. On this basis, it is essential to examine how home office affects the experience of latent benefits, which will then allow us to examine other vital organizational variables (Selenko et al., 2011).

Our aim in the present research was to learn how working from home, initiated due to the COVID-19 pandemic, affected the functions described in Jahoda’s latent deprivation model. Furthermore, we aimed to investigate its impact on other variables in organizational psychology, especially work commitment. We focused on the affective commitment component of organizational commitment, since the most recent studies have shown that emotion-based commitment is the strongest and that, if it weakens, it endangers the organization.

This approach is innovative in several respects. Primarily, we wanted to explore the latent benefits of working in a new context. Instead of focusing on a sample of unemployed people, we looked at how latent benefits change in a home office context. To our knowledge, this was a novel application of Jahoda’s model.

## Materials and methods

### Analysis design and hypotheses

We conceptualized the research question in the form of a serial multiple mediation model (see Supplementary Figure 1), where the effect of time spent working from home (*X*) on affective commitment (*Y*) is mediated by the latent functions social contact (*M*_1_) and collective purpose (*M*_2_). Although arranging the variables in a path model implies a causal description of their relationship, we emphasize that the data underlying the analysis are cross-sectional, thus we were not able to test causality. Path modeling is typically accompanied by this caveat (see Hayes, 2013, chapters 1 and 4 for a detailed discussion in the context of social psychology research); indeed, the attribution of causality is an issue related to research design and logic, and not to statistical inference (Cohen, 1988). Accordingly, the present analysis derives hypotheses consistent with theory and tests whether the collected data are consistent with the underlying argument. Furthermore, as our *a priori* hypotheses did not include the remaining latent functions, we do not analyze their relationship with the focal predictor *X* and outcome *Y*.

We hypothesized that a narrowing of social connections would lead to reduced workplace-related affective commitment if accompanied by the diminishing of collective purpose. We abbreviated the above variables in the tables and figures as TIME (*X*, the percentage of working hours spent by employees in home office in the past year), AFFECTIVE (*Y*, the extent of affective commitment measured using the Organizational Commitment Questionnaire), SOCIAL (*M*_1_, the social contact function in Jahoda’s latent deprivation model), and COLLECTIVE (*M*_2_, the collective purpose function in Jahoda’s latent deprivation model).

Using these four variables, we formulated the following hypotheses (H1–H6) to answer the research question.

*H1*: Time spent working from home (TIME) is negatively related to social contact (SOCIAL); a larger proportion of time spent working from home is associated with a decrease in social contact.

*H2*: Time spent working from home (TIME) is negatively related to collective purpose (COLLECTIVE).

*H3*: The (negative) effect of time spent working from home (TIME) on collective purpose (COLLECTIVE) is mediated through social contact (SOCIAL).

*H4*: Social contact (SOCIAL) is positively related to collective purpose (COLLECTIVE) when controlling for the effect of time spent working from home (TIME).

*H5*: The (positive) effect of social contact (SOCIAL) on affective commitment (AFFECTIVE) is mediated through collective purpose (COLLECTIVE). This hypothesis entails a positive indirect effect of SOCIAL on AFFECTIVE via COLLECTIVE, and a positive total effect of SOCIAL and AFFECTIVE.

*H6*: Collective purpose (COLLECTIVE) is positively related to affective commitment (AFFECTIVE) when controlling for time spent working from home (TIME) and social contact (SOCIAL).

### Statistical analysis

We fitted OLS regression models to each consequent variable in the structural research model depicted in Supplementary Figure 1, using the statistical software R (version 4.1.0, stats package, lm function). The regression equations, as well as direct and indirect effects, are listed in Supplementary Table 1. Each regression used the same set of complete cases (no imputed missing values). We applied parametric statistical inference for the direct effects based on unstandardized regression coefficients and the corresponding standard errors (see Supplementary Table 3), with an alpha level of 0.05 for each statistical test (exact *p* values are reported). Sobel tests were used with second-order lambda estimator for standard error to test the statistical significance of paths with single mediators (see Hayes, 2013); standard error was estimated according to Taylor et al. (2008) for the path including both mediators (Supplementary Table 4).

### Sample

Data collection was conducted by Szinapszis LLC,^1^ a healthcare market research company. Participation was voluntary and anonymous. For the data collection we used an online questionnaire. The data compilation was conducted in March and April 2021.

We asked the market research company to focus on achieving a balance between genders and age during data compilation, as it was important for our research sample to include most groups within the labor market.

There were two conditions for participation in the research: (1) respondents had to have been employed for at least 2 years at their current workplace at the time of data collection; and (2) they had to have spent at least part of their work time in home office during the period between March 2020 (the first wave of COVID-19 in Hungary) and April 2021. We successfully recruited 456 participants (239 female and 217 male). Most worked in the public sector (203) or private sector (194). Our sample showed a balanced distribution of age (in the analyzed sample, 19% were between the ages of 18 and 30; 23% were between the ages of 31 and 39; 27% were between the ages of 50 and 64; and 4% were older than 65). The research sample showed a heterogeneous distribution in terms of organization size and economic sector. Our questionnaire was filled out by employees in 14 different sectors, so we can safely say that we covered most aspects of the Hungarian labor market (most participants were from the education and cultural sectors and the service sector). Nevertheless, our sample cannot be regarded as representative of the entire Hungarian labor market.

Importantly, our research sample was restricted to employees who were able to work productively from home and who had not been forced to take leave of absence.

The study was approved by the Research Ethics Committee of the authors’ institution (reference number: blinded for the review).

### Scales

The variables in the hypotheses were measured using the following methods. Time spent working from home was measured as the self-reported proportion of work (‘Since March 2020, what percentage of your work time have you spent in home office altogether?’). We categorized responses into five ordinal categories with the following values: 0: up to 20%; 1: up to 40%; 2: up to 60%; 3: up to 80%; 4: up to 100%.

We measured the latent functions using the Latent and Manifest Benefits (LAMB) scale (Muller et al., 2005). The questionnaire comprises 36 items measuring both Jahoda’s latent benefits theory and Fryer’s manifest benefits (salary). Although this instrument is frequently used (e.g., Paul and Batinic, 2010; Selenko et al., 2011; Muller and Waters, 2012; Aitken et al., 2021), a shorter version has also been published by Kovacs. While the long version measures six factors using six items per scale, in the short version this is reduced to three items per scale (Kovacs et al., 2019). We measured five of the six factors using a seven-point Likert scale. We omitted the sixth factor from the original LAMB questionnaire (financial strain), since this question is more relevant to an unemployed than an employed sample. In our research, we used the two functions presented above: social contact (‘I usually have a lot of opportunities to mix with people’; ‘I often meet new people’; ‘I often go out and meet with others’) and collective purpose (‘I often feel that I make a meaningful contribution to society’; ‘I often feel a valuable part of society’; ‘I hold a valuable position in society’). In addition, we mean-centered these variables to support the interpretation of regression coefficients. At this time, the LAMB scale did not have a validated Hungarian translation. Both scales had a satisfactory internal consistency (Cronbach’s alpha): SOCIAL = 0.88; COLLECTIVE = 0.92.

We used the Hungarian version (Kiss et al., 2012) of the Organizational Commitment Questionnaire (Meyer and Allen, 1991). The questionnaire is based on the three-component TCM Employee Commitment Survey and uses a six-point Likert scale to measure the subscales, with 12 items altogether (Jaros, 2007). The scale of affective commitment (e.g., ‘I would be very happy to spend the rest of my career with this organization’) had satisfactory internal consistency, with a Cronbach’s alpha value of.94.

The analyzed datasets are available in the Open Science Framework repository: https://osf.io/f4ern/?view_only=67dceb3cbcf74c33923143e06ec43578.

## Results

The descriptive statistics for each modeled variable are presented in Supplementary Table 2. The results of the hypotheses tests are summarized in Supplementary Figure 2. Supplementary Table 3 presents the parameters of the research model; Supplementary Table 4 presents the indirect effects of time spent working from home (*X*) on affective commitment (*Y*) through the mediators (*M*_1_ and *M*_2_); and Supplementary Table 5 summarizes the effects of social contact (*M*_1_) on affective commitment (*Y*).

Hypothesis 1 was supported: more time spent working from home was negatively related to social contact. Specifically, one unit increase in TIME (approximately 20% more time spent working from home) was associated with a 0.2 unit decrease in social contact (measured 1–7).

Hypothesis 2 was refuted: more time spent working from home was not associated with a decrease in collective purpose. However, this hypothesis was related to the total effect of TIME on COLLECTIVE (i.e., the sum of its direct effect and its indirect effect through SOCIAL). Indeed, in support of Hypothesis 3, the (negative) effect of time spent working from home on collective purpose was mediated through social contact, with a statistically significant indirect effect, a_1_d_21_ = −0.081, SE = 0.020, *z* = −4.011, *p* < .001.

Hypothesis 4 was supported: social contact was positively related to collective purpose when controlling for the effect of time spent working from home: one unit increase in social contact led to a 0.4 unit increase in collective purpose (both measured 1–7).

Hypothesis 5 was supported: the effect of social contact on affective commitment was fully mediated through collective purpose (note that the hypothesis was related to the indirect and total effects, not the direct effect). The total effect of SOCIAL on AFFECTIVE (controlling for the effect of TIME) was 1.9: one unit increase in social contact (measured 1–7) was associated with a 1.9 unit increase in affective commitment (measured 1–6).

Hypothesis 6 was supported: collective purpose was positively related to affective commitment when controlling for the effects of time spent working from home and social contact. One unit increase in COLLECTIVE (measured 1–7) was associated with a 0.28 unit increase in AFFECTIVE (measured 1–6).Time spent working from home (*X*) had a statistically significant, positive direct effect on affective commitment (*Y*) when controlling for the effects of latent functions, albeit a small one. Notably, the indirect effect of time spent working from home through both latent functions was statistically significant and negative (see Supplementary Table 4): less time spent at the workplace led to less favorable latent deprivation outcomes, which, in turn, were positively related to affective commitment. The total effect of time spent working from home was statistically significant and positive, although small in magnitude, *b* = 0.080, *p* = 0.043; indeed, it explained less than 1% variance in affective commitment (*R*^2^ < 0.009). Conversely, together with the latent functions, it explained over 13% variance in affective commitment. Together, these findings indicate that more time spent working from home is not expected to lead to reduced affective commitment to the workplace if there is compensation for latent functions that are themselves prone to be negatively affected by more time spent working from home. Of the two functions in the research model, collective purpose had a bigger impact on affective commitment, which showed no systematic decrease with more time spent working from home (Hypothesis 2 refuted), despite social contact being negatively affected by it (also see Supplementary Table 2 for the mean values of the functions at different levels of TIME).

### Discussion, limitations, and future work

The COVID-19 pandemic has resulted in many changes in every field of life. We have had to face tragedies, economic damage, and permanent change both as individuals and as a society. The pandemic has had a major impact on consumer behavior (Gajić et al., 2022) and on the world of work (de Alves et al., 2021). While some employees have lost their jobs and some organizations have gone bankrupt (Nicola et al., 2020), other organizations have begun to adopt new approaches. With the development of home office, work has slowly shifted from offline to cyberspace. Digitalization, a process of renewal that began decades ago, has reached industries, organizations and jobs in a way that would have been impossible if not for the pandemic (de Alves et al., 2021). The rapid spread of the home office has created a new psychological situation, unfamiliar to managers, employees, and researchers. Working from home differs from traditional offline work. Its structure, benefits, and disadvantages could not be more different (Narbarte et al., 2020), thus we cannot be sure of the kind of effects it will have on organizational psychological factors such as organizational commitment.

Organizational commitment, and within this affective commitment, is a psychological factor that can significantly affect an organization’s performance. In the case of high affective commitment, employees not only connect emotionally with the organization but also accept its values (Somers, 1995). As a result, fewer employees go on sick leave and staffing fluctuations are reduced, while at the same time workplace motivation and additional workload will increase (Solinger et al., 2008; Alkhateri et al., 2018). Understandably, all organizations aim to cultivate high affective commitment among their employees, making this an essential task for managers (Mercurio, 2015). Organizational commitment can be influenced by various factors, such as employee satisfaction (Matzler and Renzl, 2007) and job satisfaction. However, to date no research has focused on the impact of latent benefits on organizational commitment.

According to the latent deprivation model proposed by Jahoda (1982), work has not only manifest benefits, such as salary, but also latent benefits. She defines five such latent benefits. According to Jahoda, when a person loses their job, the loss of these latent benefits will have an adverse effect on their well-being (Jahoda, 1982). Many earlier studies have examined Jahoda’s model in the context of the unemployed population (e.g., Waters and Moore, 2002; Paul and Batinic, 2010; Selenko et al., 2011), while others have focused on the retired population (Read et al., 2013), people who are out of the labor force (Paul et al., 2009), voluntary workers (Yang and Matz, 2020), and low-status employees (Batinic et al., 2010). However, to date no one has studied the latent benefits of work on employees working from home. The innovative aspect of our research lies in the fact that our aim was to approach Jahoda’s model from a new perspective. It is essential to point out that working from home has been a method of work organization for 40 years. What is unprecedented is the high number of employees who are now obliged to work from home. It is therefore essential to examine how the experience of previously taken-for-granted latent benefits has been affected. During our research, we focused on the impact of the amount of time spent working from home on affective commitment by examining latent functions. We approached our research questions using the serial multiple mediation model. The resulting model (Supplementary Figure 2) proves that there is a negligible direct connection between the amounts of time spent working from home and affective commitment. However, if we look at social contact and collective purpose (as the latent function perspective), we observe a significant connection. The explanation behind this is that the more time an employee spends away from their workplace, the harder it becomes for them to maintain their social relationships, thus from a social perspective, their relationships are narrowed and they receive less information, and the negative impacts of this cannot be compensated by the extra time they spend with their family (Jahoda, 1982). Social relationships have a moderately strong, positive connection to collective purpose (i.e., how useful and beneficial the employee thinks their job is). Since the time spent working from home weakens social contact, the experience of collective purpose will also be affected, leading to a decline in affective commitment. Therefore, together with the two latent functions, it explained over 13% variance in affective commitment. The decline in affective commitment may increase staff fluctuation and absences and lower performance and organizational citizenship behaviors, which will have a negative impact on the organization (Solinger et al., 2008; Wołowska, 2014).

We intended our model as a contribution to the slowly emerging picture of the effects of the COVID-19 pandemic on organizational commitment. Based on previously published studies, organizational resilience (Filimonau et al., 2020), organizational support (Alshaabani et al., 2021), and pandemic-induced stress (Kang et al., 2021) can be said to have significantly impacted organizational commitment during the pandemic. Other researchers have pointed out that affective commitment influences organizational psychological variables, such as employees’ knowledge application behavior (Ishak et al., 2022) and organizational creativity (Mohammed et al., 2022). The results of our research further highlight how organizational affective commitment is a highly complex notion that depends on many organizational variables, thus organizations and employers need to gain a more profound knowledge and ensure the provision of favorable conditions.

We would like to point out that working from home and digitalization are integral and essential aspects of the world of work. Working from home has various benefits, although many authors warn against its dangers (Davis et al., 2020; Gibbs et al., 2021; Howe and Menges, 2021). Since home office will remain even after the COVID-19 pandemic is over, it is essential that organizations and managers be given a comprehensive picture of what can be done for their employees’ psychological well-being and effectiveness (Sears et al., 2013). One of the main findings is that when employees work from home, some of the latent functions that are taken for granted are negatively affected, which may have significant psychological consequences for the employee. Based on our model, we believe that managers must aim to provide opportunities for home office workers to experience the social advantages of their workplace. It might be beneficial to limit the amount of time spent working from home; to offer the possibility of a hybrid work schedule (e.g., 3 days a week in home office and 2 days in the office); and to provide opportunities for the social validation of employees’ contribution to work goals. Another possibility would be to organize events outside working hours, where social connections could be enhanced. It would also be helpful to create a digital space where employees who are working from home can keep in touch and reflect on other people’s achievements. Another useful idea would be to highlight the significance of the work, thereby strengthening the sense of collective purpose.

Our research was complicated by certain limitations. The research was carried out in the form of a cross-sectional study using a self-administered online questionnaire, thus the respondents’ subjective distortions (e.g., the time they spent working from home in percentages) may have influenced our results. Although we asked respondents to concentrate on their experiences in home office when filling out the LAMB questionnaire, this partly happened retrospectively. Also, the LAMB scale had no validated Hungarian translation at this time. Our sample cannot be regarded as representative of the entire Hungarian labor market, and there was no balance in terms of the 14 examined sectors among the people who filled out our questionnaire. The results of our research are further limited by the effects of the COVID-19 pandemic at the social level, which could not be included in our present examination. It is possible that the impact on affective commitment was also influenced by non-organizational psychological influences such as the pandemic-induced collective sense of grieving and loss, and large-scale mental health issues. Furthermore, our questionnaire did not examine how much of the employee’s work time was spent in video calls, meetings, or phone calls, which affect the experience of social contact. In future research, it would be beneficial to examine how the use of specific digital devices and channels can reduce social alienation and its negative impact on affective commitment.

In the future, it would be worth examining the effects of the latent functions on affective commitment not just in a cross-sectional study but also using a longitudinal study design. Moreover, the research could be expanded to other major organizational psychological factors, such as work motivation, organizational trust, job satisfaction, and productivity to examine how home office employees have been affected. It would also be worth exploring the kind of help offered to home office employees by organizations and managers in an effort to improve the experience of the latent benefits of work, and how effective these methods are. The findings could contribute to the development of long-term programs of assistance and intervention.

## Conclusion

We analyzed the role of latent functions in promoting affective organizational commitment while working from home. Although the proportion of time spent working from home was not directly related to affective commitment, we demonstrated, using a multiple serial mediator path model, that its effect was mediated by latent functions. Specifically, we found that more time spent in home office was associated with a decrease in social contact, the effect of which on affective commitment was mediated through collective purpose. Thus, promoting the perception of collective purpose in employees is expected to mitigate the negative effect of a narrowed social sphere resulting from an increased proportion of time spent in home office.

## Data availability statement

The original contributions presented in the study are included in the article/Supplementary material, further inquiries can be directed to the corresponding author.

## Ethics statement

The studies involving human participants were reviewed and approved by Research Ethics Committee of Eötvös Loránd University, Faculty of Education and Psychology (ELTE PPK; Reference number: 2021/222, Date: 26. 04. 2021). The patients/participants provided their written informed consent to participate in this study.

## Author contributions

AS, KF, OP, and OK contributed to conception and design of the study. AS, KF, and OK organized the database. GA performed the statistical analysis and wrote sections of the manuscript. AS wrote the first draft of the manuscript. All authors contributed to the article and approved the submitted version.

## Funding

This research was conducted in the framework of the “Doctoral Projects 2020/2021” syndicated research funding scholarship of Eötvös Loránd University’s Faculty of Education and Psychology.

## Conflict of interest

The authors declare that the research was conducted in the absence of any commercial or financial relationships that could be construed as a potential conflict of interest.

## Publisher’s note

All claims expressed in this article are solely those of the authors and do not necessarily represent those of their affiliated organizations, or those of the publisher, the editors and the reviewers. Any product that may be evaluated in this article, or claim that may be made by its manufacturer, is not guaranteed or endorsed by the publisher.

## Supplementary material

The Supplementary material for this article can be found online at: https://www.frontiersin.org/articles/10.3389/fpsyg.2022.1002818/full#supplementary-material

## Footnotes

^1^ https://szinapszis.hu/en
