# Supplementary material for: The impact of time spent working from home on affective commitment in the workplace: The mediating role of social relationships and collective aims
Source: Front Psychol. 2023 Jan 13;13:1002818. doi: 10.3389/fpsyg.2022.1002818 (PMC9880430; doi:10.3389/fpsyg.2022.1002818)
Supplement: Supplementary file 1 [file Data_Sheet_1.docx]

**Appendix**

(Figure 1 Path diagram of the research model and the corresponding hypotheses)
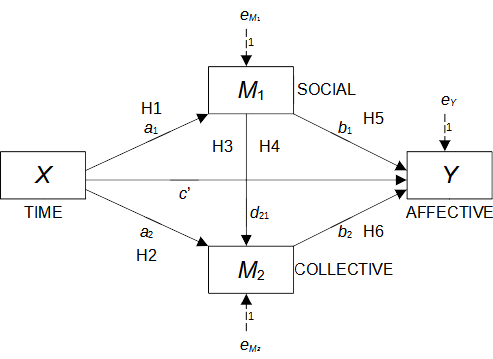


(Table 1. A summary of hypotheses, indirect effects of X on Y, and the corresponding regression equations).

| Research hypotheses | | | |
| --- | --- | --- | --- |
| Abb. | Path | Coefficient(s) | Text |
| H1 | $X\to M_{1}$ | $a_{1}$ | TIME is negatively related to SOCIAL |
| H2 | $X\to M_{2}$ | $a_{2}+a_{1}d_{21}$ | TIME is negatively related to COLLECTIVE |
| H3 | $X\to M_{1}\to M_{2}$ | $a_{1}d_{21}$ | TIME to COLLECTIVE through SOCIAL |
| H4 | $M_{1}\to M_{2}.X$ | $d_{21}$ | SOCIAL to COLLECTIVE given TIME |
| H5 | $M_{1}\to Y.M_{2}X$ | $b_{1}$ | SOCIAL to AFFECTIVE given all else |
| H6 | $M_{2}\to Y.M_{1}X$ | $b_{2}$ | COLLECTIVE to AFFECTIVE given all else |
| Indirect effects of X on Y | | | |
| Ind1 | $X\to M_{1}\to Y$ | $a_{1}b_{1}$ | TIME to AFFECTIVE through SOCIAL |
| Ind2 | $X\to M_{2}\to Y$ | $a_{2}b_{2}$ | TIME to AFFECTIVE through COLLECTIVE |
| Ind3 | $X\to M_{1}\to M_{2}\to Y$ | $a_{1}d_{21}b_{2}$ | TIME to AFFECTIVE through both mediators |
| Regression equations | | | |
| Eq1 | $M_{1}=i_{M_{1}}+a_{1}X+e_{M_{1}}$ | | SOCIAL on TIME |
| Eq2 | $M_{2}=i_{M_{2}}+a_{2}X+d_{21}M_{1}+e_{M_{2}}$ | | COLLECTIVE on TIME and SOCIAL |
| Eq3 | $Y=i_{Y}+c^{'}X+b_{1}M_{1}+b_{2}M_{2}+e_{Y}$ | | AFFECTIVE on all |

*Notes*. *X* = TIME, *M*_1_ = SOCIAL, *M*_2_ = COLLECTIVE, and *Y* = AFFECTIVE.

(Table 2. Descriptive statistics of the outcome and mediator variables at different levels of proportion of time spent in home office.)

|  |  | *Y*  AFFECTIVE | *M*_1_  SOCIAL | *M*_2_  COLLECTIVE | *Y*  adjusted |
| --- | --- | --- | --- | --- | --- |
| Up to 20% (X = 0) | Mean | 4.081 | 3.700 | 4.485 | 5.497 |
| *n* = 121 | *SD* | 1.248 | 1.541 | 1.361 |  |
| 21-40% (X = 1) | Mean | 3.860 | 3.959 | 4.760 | 5.681 |
| *n* = 89 | *SD* | 1.303 | 1.565 | 1.304 |  |
| 41-60% (X = 2) | Mean | 4.140 | 3.887 | 4.710 | 5.748 |
| *n* = 77 | *SD* | 1.177 | 1.356 | 1.208 |  |
| 61-80% (X = 3) | Mean | 4.125 | 3.292 | 4.625 | 5.763 |
| *n* = 64 | *SD* | 1.305 | 1.511 | 1.392 |  |
| 81-100% (X = 4) | Mean | 4.362 | 2.952 | 4.686 | 5.840 |
| *n* = 105 | *SD* | 1.315 | 1.484 | 1.570 |  |
| Total | Mean | 4.118 | 3.553 | 4.643 |  |
| *n* = 456 | *SD* | 1.276 | 1.542 | 1.380 |  |

*Note*. The corresponding total means are added to *M*_1_ and *M*_2_ above to reverse mean-centring. The adjusted values of Y are generated using Eq3 presented in Table 1, using the mean values of each mediator at different levels of X.

(Figure 2. The effect of time spent in home office (X) and latent-deprivation factors (M1: social contacts; M2: collective goal) on affective commitment (Y). )


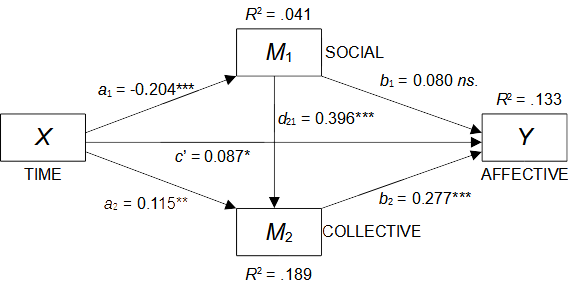


(Table 3. Regression parameters and model summary of the research model.)

|  | Consequent | | | | | | | | | | | |
| --- | --- | --- | --- | --- | --- | --- | --- | --- | --- | --- | --- | --- |
|  |  | *M*_1_ (SOCIAL) | | |  | *M*_2_ (COLLECTIVE) | | |  | *Y* (AFFECTIVE) | | |
| Antecedent |  | Coeff. | *SE* | *p* |  | Coeff. | *SE* | *p* |  | Coeff. | *SE* | *p* |
| X (TIME) | *a*_1_ | -0.204 | 0.047 | < .001 | *a*_2_ | 0.115 | 0.039 | .004 | *c'* | 0.087 | 0.040 | .023 |
| *M*_1_ (SOCIAL) |  | --- | --- | --- | *d_2_*_1_ | 0.396 | 0.039 | < .001 | *b*_1_ | 0.080 | 0.041 | .052 |
| *M*_2_ (COLLECTIVE) |  | --- | --- | --- |  | --- | --- | --- | *b*_2_ | 0.277 | 0.045 | < .001 |
| Constant | *i*_M1_ | 0.383 | 0.113 | < .001 | *i*_M2_ | -0.215 | 0.094 | .022 | *i*_Y_ | 3.956 | 0.090 | < .001 |
|  |  |  |  |  |  |  |  |  |  |  |  |  |
|  |  | *R*^2^ = .041 | | |  | *R*^2^ = .189 | | |  | *R*^2^ = .133 | | |
|  |  | *F*(1, 454) = 19.18, *p* < .001 | | |  | *F*(2, 453) = 52.87, *p* < .001 | | |  | *F*(3, 452) = 23.01, *p* < .001 | | |

(Table 4. Indirect effects of X (TIME) on Y (AFFECTIVE) in the research model.)

| Abb. | Path | Coeffs. | Effect | *SE* | *z* | *p* |
| --- | --- | --- | --- | --- | --- | --- |
| Ind1 | $X\to M_{1}\to Y$ | $a_{1}b_{1}$ | -0.016 | 0.009 | -1.745 | .081 |
| Ind2 | $X\to M_{2}\to Y$ | $a_{2}b_{2}$ | 0.032 | 0.012 | 2.616 | .009 |
| Ind3 | $X\to M_{1}\to M_{2}\to Y$ | $a_{1}d_{21}b_{2}$ | -0.022 | 0.007 | -3.371 | < .001 |

*Note*. For Ind1 and Ind2, we used a Sobel test with second-order lambda estimator for standard error (see Hayes, 2013); for Ind3, we estimated standard error according to Taylor et al., (2008)

(Table 5. The effect of M1 (SOCIAL) on Y (AFFECTIVE).)

|  | Path | Coeffs. | Effect | *SE* | Statistic | *p* |
| --- | --- | --- | --- | --- | --- | --- |
| Direct | $M_{1}\to Y.M_{2}X$ | $b_{1}$ | 0.080 | 0.040 | *t*(452) = 1.952 | .052 |
| Indirect ^a^ | $M_{1}\to M_{2}\to Y.X$ | $d_{21}b_{2}$ | 0.110 | 0.021 | *z* = 5.263 | < .001 |
| Total ^b^ | $M_{1}\to Y.X$ |  | 0.190 | 0.039 | *t*(453) = 4.936 | < .001 |

^a^: Statistical inference for the indirect effect is done by a Sobel test with second-order lambda estimator for standard error.

^b^: The total effect is the sum of the direct and indirect effects; statistical inference is done by regressing Y onto M1 and X with M2 absent from the model.
